# Genetic Composition of Polish Hucul Mare Families: mtDNA Diversity

**DOI:** 10.3390/genes15121607

**Published:** 2024-12-17

**Authors:** Aleksandra Błaszczak, Monika Stefaniuk-Szmukier, Bogusława Długosz, Adrianna Dominika Musiał, Katarzyna Olczak, Katarzyna Ropka-Molik

**Affiliations:** 1 Department of Animal Reproduction, Anatomy and Genomics, University of Agriculture, al. Mickiewicza 24/28, 30-059 Cracow, Poland; aleksandra.blaszczak@iz.edu.pl (A.B.); boguslawa.dlugosz@urk.edu.pl (B.D.); 2 Department of Animal Molecular Biology, National Research Institute of Animal Production, Krakowska 1, 32-083 Balice, Poland; adrianna.musial@iz.edu.pl (A.D.M.); katarzyna.ropka@iz.edu.pl (K.R.-M.); 3 Department of Farm Animal Biodiversity Conservation and Horse Breeding, National Research Institute of Animal Production, Krakowska 1, 32-083 Balice, Poland; katarzyna.olczak@iz.edu.pl

**Keywords:** mtDNA, genetic resources, Hucul horse, population genetics

## Abstract

**Backround:** The Hucul horse breed formed in the region of the Eastern Carpathians, likely through the natural crossbreeding of oriental horses. After World War II, their population significantly decreased, leading to the breeding being based on only 14 female lines, whose founders often had unknown origins. To preserve the breed’s unique characteristics, it is now part of a Genetic Resources Conservation Program, which prioritizes the maintenance of genetic diversity. This study aims to clarify the maternal relatedness of founder mares and assess genetic diversity using mitochondrial DNA (mtDNA). **Methods:** The hyper-variable region of the mitochondrial genome was analyzed in 57 horses. Pedigree records were used to trace genealogical lines, and molecular analysis focused on identifying maternal relationships between founder mares. **Results:** The analysis revealed close maternal kinships between the lines of Jagoda and Bajkałka, as well as Sekunda and Sroczka. In the Hucul population, seventeen mitochondrial haplotypes were identified, with three that did not match any established lines. The findings reveal discrepancies between pedigree records and mitochondrial DNA data, suggesting potential inaccuracies in the Hucul horse studbook. **Conclusions:** The findings highlight the importance of combining pedigree and molecular data to refine strategies to preserving genetic diversity, minimizing inbreeding, and improving the management the Genetic Resources Conservation Program.

## 1. Introduction

For centuries, thousands of animal species have gone extinct, and one-third of all domestic animal species are threatened. According to data from the Food and Agricultural Organization (FAO), 284 horse breeds are at risk of extinction. This group includes Hucul horses, for which maintaining the breed and preserving genetic diversity is a priority. The more founders a given population has, the longer it is capable of genetic renewal. Through pedigrees, we can trace the genealogical lines of mares and stallions, but molecular studies, particularly mtDNA analysis, allow for the precise identification of the founding mare lineage groups and provide information about their relatedness in the female line. In this way, the protection of genetic diversity can become more reliable [1].

The Hucul horse breed developed in the Eastern Carpathians, likely through natural crossbreeding of Arabian, Tatar, Turkish, Przewalski, Noriker, and Asian horses. The earliest mention of Hucul horses dates back to 1603 in the publication Hippica by Krzysztof Dorohostajski, where they were described as excellent mountain horses capable of thriving in harsh conditions [2]. The first Hucul horse stud was established in 1856 in Luczyn, within the then Austro-Hungarian Empire. At that time, the horses were used by the military as sumpter animals. During World War I, many horses were taken into the army, and while under occupation, they were crossbred with Russian stallions. In 1919, the Luczyn Stud was reactivated, where mares were also bred with Arabian stallions. In Poland, during the interwar period, only two Hucul horse studs existed, managed by Stanisław Mencel in the Pawełcze estate and in Niskołyzy [3]. In 1924, 323 mares were found during registration, and a year later, the Association of Hucul Horse Breeders was established, which maintained the studbook that recorded 418 mares [2].

After World War II, their population significantly decreased. In Poland, three mares and two stallions were found in state breeding farms. To restore the population, twelve mares and one stallion of Hungarian origin were brought from Germany to Poland as part of the post-war restitution. Additionally, six mares, mostly undocumented, were found in private breeding. Around 1946, the Hucul horse stud was established in Kwilicz, where all Hucul mares were gathered. Several relocations of the breeding center hindered breeding efforts. The horses were settled more permanently in 1958 at the Siary Stud, although many had one-sided or incomplete pedigrees [2,4]. Since then, breeding has been conducted to preserve the purity of the breed.

As one of Poland’s oldest horse breeds with a small population, the Hucul horse is included in the Genetic Resource Protection Program (GRPP), to maintain and increase the population while preserving genetic variability, a stable breed standard, and improving functional traits without changing the horse type [5,6]. The Hucul Horse Stud Book (HHSB) currently lists horses belonging to 14 female and 7 male bloodlines. In zootechnical nomenclature, a family is defined as the offspring of a specific female ancestor [7]. The families among the 14 female lines of Hucul horses are Agatka, Bajkałka, Czeremcha, Wrona, Gurgul, Jagoda, Laliszka, Nakoneczna, Wołga, Polanka, Sroczka, Sekunda (Żyrka), Wydra, and Reda. According to the first volume of the HHSB, three more mares started their own families: Cyrla, Gostka, and Kukułka, but these families have completely disappeared [2,8,9].

The identification of horses based on external traits has been known for centuries. Easily distinguishable features included sex and coat color [10]. Later, horse branding, freeze marking, and photography were introduced [11]. Technological advancements enabled new identification methods, such as microchips that identify specific individuals but do not provide lineage information [12]. Currently, genetic markers such as microsatellites, minisatellites, and SNPs are used to verify ancestry (mandatory checks during licensing or for foals in Hucul horses since 2007 [13]). New techniques using mitochondrial DNA (mtDNA), which is passed down exclusively through the maternal line, allow for the creation of phylogenetic trees and the determination of family origins [14].

The mtDNA in equines is a circular genome of 16,660 base pairs, consisting of 13 protein-coding genes without introns, which are part of five protein complexes. It also includes 22 transfer RNAs, 2 ribosomal RNAs, and the control region (D-loop or displacement region). The D-loop is approximately 1200 base pairs long and contains regulatory elements for replication and transcription. It also features two hypervariable regions (HVR1 and HVR2), a large conserved sequence block, and three smaller conserved sequence blocks with tandem repeats of an 8 bp equine-specific sequence (TGTGCACC) [15].

As the genetic resources protection program is still being actively implemented, there is a need to monitor genetic diversity. This study aimed to examine the kinship between mare families and determine whether a pedigree analysis is sufficient to assess the number of individuals in each maternal line. The use of mtDNA analysis would be a crucial first step in identifying which maternal lines are endangered and require greater attention to maintain genetic diversity in the small Hucul horse population.

## 2. Materials and Methods

### 2.1. Ethical Statement

Peripheral blood and hair follicle samples were obtained for routine horse parentage testing or hair follicles and blood samples that came from a genetic material bank of the National Research Institute of Animal Production, Poland. Each owner was informed about how the data concerning their horses would be used in the study, understood this information, and voluntarily consented to its use.

### 2.2. Samples, DNA Extraction, and Sanger Sequencing

The Hucul Horse Studbook (Khc) includes horses belonging to 7 male lines and 14 female lines. Despite the observed growth trends, the current population size remains small, considering the distribution of individuals across the recognized female lines. Based on the number of mares recorded in the studbooks and the calculated risk status index, it has been determined that the Hucul breed requires continued conservation efforts.

Pedigree data were obtained from volumes of the Hucul Horse Studbook, excluding mares that do not belong to the 14 officially recognized Polish female lines. Ultimately, the dataset encompassed over 8000 female individuals born between 1930 and 2022.

Based on pedigree analyses 57 horses from 14 maternal lines included in GRPP (Agatka—3, Bajkałka—3, Czeremcha—4, Wrona—4, Gurgul—3, Jagoda—4, Laliszka—6, Nakoneczna—3, Wołga—7, Polanka—4, Sroczka—4, Sekunda—2, Wydra—7, Reda—3) were selected while maintaining the greatest possible genetic distance in the female line. The lineage of the horses was determined using pedigree data available in the Polish Horse Breeders Association database [16]. The samples used for this study, consisting of hair follicles and blood, were obtained from the genetic material bank of the National Research Institute of Animal Production in Poland.

The DNA was isolated using the Sherlock AX kit (A&A Biotechnology, Gdańsk, Poland) according to the provided protocol. The quality of the isolated samples was measured using the NanoDrop 2000 spectrophotometer (Thermofisher Scientific, Waltham, MA, USA).

To conduct Sanger sequencing of hypervariable regions 1 and 2 of the mitochondrial DNA D-Loop were amplified using primers: F: AACGTTTCCTCCCAAGGACT and R: GTAGTTGGGAGGGTTGCTGA, HVR1B F: ACCCCATCCAAGTCAAATCA and R: CAGGTGCACTTGTTTCCTATG, HVR2 F: ACCTACCCGCGCAGTAAGCAA and R: ACGGGGGAAGAAGGGTTGACA [14] based on the reference sequence NC_001640 [17]. The HVR1A and HVR1B primers amplify 716 bp of HVR1 and 263 bp of HVR2, covering a total of 979 bp out of the 1200 bp complete D-loop sequence. The unsequenced non-hypervariable region (positions 16,137–16,357) was reconstructed by complementing it with the reference sequence. The amplification region between positions 15,542 and 16,611 (length 1069 bp) was carried out using the Phanta Ready Mix (Vazyme Biotech, Nanjing, China) and AmpliTaq Gold 360 Master Mix (Life Technologies, Carlsbad, CA, USA) at an annealing temperature of 56 °C. To purify the obtained products after the PCR reaction from free nucleotides and primers, the EPPiC enzyme mixture (A&A Biotechnology, Gdańsk, Poland) was used according to the protocol. Sequencing PCR was performed using the BigDye™ Terminator v3.1 Cycle Sequencing Kit (Life Technologies, Carlsbad, CA, USA). The products were then purified using the BigDye X-Terminator Purification Kit (ThermoFisher Scientific, Carlsbad, CA, USA) according to the protocol. Sanger sequencing was conducted on the Genetic Analyzer 3500x1 (Applied Biosystems, Waltham, MA, USA).

### 2.3. Data Analyses

**Figure 1:**
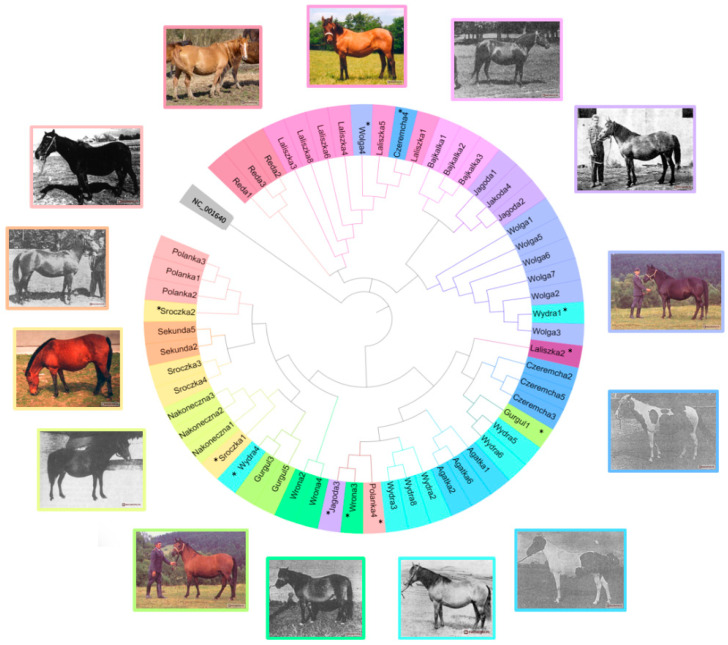
The circular neighbor-joining tree, with each horse represented by color and named according to the dam line information from the pedigree. Individuals with errors in the pedigree are marked with *. Next to the diagram, the oldest available photos of representatives of the female lines were placed, framed in the color corresponding to each line. The neighbor-joining tree was constructed using the Mega 11 software and edited in iTOL (available online: https://itol.embl.de) and PowerPoint.

**Figure 2:**
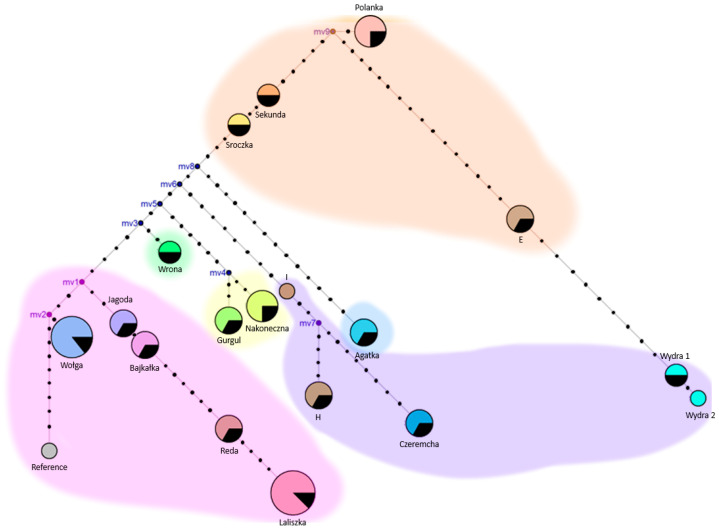
The median-joining network of the identified Hucul horse haplotype and reference (NC_001640). Node sizes represent haplotype frequencies; partially black fragment represents contribution of one individual. If there is no black color, only one individual represents this haplotype. Mv1-9 are median vectors. The colors of highlighted areas refer to haplogroups identified by Jansen et al. (2002) [20]: pink indicates haplogroup A, green—B, purple—C, yellow—D, orange—F, and blue—G. Strokes on the branches correspond to the number of polymorphisms.

The raw files were read using FinchTV 1.3.0 (Geospiza, Inc. Seattle, WA, USA). The results were compared with each other and with the reference sequence (GenBank NC_001640) in Mega 11 software [18]. The unsequenced fragment (16,137–16,357) containing tandem repeats was supplemented according to the reference sequence. The compilation of results was exported and processed in Excel 2016 (Microsoft, Redmond, WA, USA). A pie chart (Figure 1) was created using Mega 11 and then edited in iTOL (Available online: https://itol.embl.de (accessed on 11 January 2024)). The neighbor-joining phylogenetic tree (Figure S1) was constructed in Mega 11. A median-joining network of haplotypes (Figure 2) was also constructed using Network 4.6.1.6 software, with editing performed in GIMP 2.10.34 (The GIMP Development Team, Orinda, CA, USA) and PowerPoint 2016 (Microsoft, Redmond, WA, USA). Average number of nucleotide differences and nucleotide diversity were calculated using DnaSP v.6 [19].

## 3. Results

After examining 57 Hucul horses from 14 families, 17 haplotypes were identified, including 1 in 2 variants. Thirteen horses (22.8%) among the studied individuals belonged to a different family than indicated by the pedigree analysis (Figure 1). All sequences have been deposited in GenBank under accession numbers PP002986-PP003003. Within the D-loop region, 63 polymorphic sites were identified, including 2 characteristic of all representatives of this breed, at positions 15,495 and 15,720 in comparison to the reference sequence (Table 1A–C). An analysis was performed in the DnaSP program, including nucleotide diversity (π = 0.015) and the average number of nucleotide differences (k = 17.404).

A haplotype classification based on the HVR1 fragment in both domesticated and wild horses (including material from archaeological sites) was created, identifying six groups: A, B, C, D, F, and G [20] The structure analysis based on mtDNA haplotype frequency showed that Polish Hucul horse families also belong to these groups (Figure 2). Most families belong to group A (29.4%); these include the Laliszka, Reda, Bajkałka, Jagoda, and Wołga families. The Wrona family is the sole representative of haplogroup B (5.9%). Group C (23.5%) is represented by the Wydra, Czeremcha, and haplotypes H and I, group D (11.8%) by the Nakoneczna and Gurgul families, group F (23.5%) by Sroczka, Sekunda, Polanka, and haplotype E, and group G (5.9%) by the Agatka family. Analyzing the population of Hucul horses in the Czech Republic [16], haplogroup A was also the most represented (46.6%), followed by group F (20%). Group B comprised 13.3% of the maternal lines, while groups C, D, and E each represented 6.7%.

The analysis of the phylogenetic tree (Figure 1) revealed four distinct branches grouping closely related families. The first branch includes the families of Reda (haplotype Z) and Laliszka (L). The second branch contains the family of Wołga (haplotype O), Jagoda (J), and Bajkałka (B). The third branch consists of the Czeremcha (C) along with haplotypes I and H, which do not belong to any family, Agatka (A), the Wydra family (W1 and W2), and haplotype E. The final branch represents the families of Sroczka (S), Sekunda (T), Polanka (P), Wrona (F), Nakoneczna (N), and Gurgul (G). The closest relationship was observed between the Bajkałka and Jagoda families.

In the median-joining network of the identified Hucul horse haplotypes (Figure 2), it is shown how haplotypes transformed into one another or split apart. The most primitive haplotype represents the Wołga family. From this point, we can observe the emergence of subsequent polymorphisms, marked as black strokes on the branches, leading to the formation of new haplotypes that exist to this day. Sometimes, evolution proceeded in both directions. In such cases, we expect a common ancestor indicated as mv1-9 (median vectors—hypothetical (often ancestral) sequences that are required to connect existing sequences within the network), which gave rise to further mutations and haplotypes.

## 4. Discussion

It is believed that the Hucul horse breed was shaped in the Eastern Carpathians under the influence of Oriental horses, Przewalski horses, and Noriker horses [21]. Differences in the proportional presence of these maternal lines obtained within the presented study may suggest how mares migrated as the breed expanded beyond the Eastern Carpathians.

Little is known about the 14 founder mares (Figure 1). Laliszka, bred by Stanisław Stawiarski in 1945, might have been crossbred with a Malopolski horse. Reda’s birth year is unknown, but she had her first foal in 1961. Bajkałka, born in 1944, was bred by Stanisław Kozioł. Jagoda, born in 1937, was bred by Jakub Twardowski. On her father’s side, she had a documented pedigree for three generations, while on her mother’s side, her mother Basia and grandmother Góralka Nowosądecka appeared twice in the father’s pedigree. Wołga was born in 1936, Wrona in 1934, and Wydra in 1929, all three from Hungary, and after World War II, were brought to Poland from Germany as part of restitution, without documentation, and placed in Kwilicz. The mare Czeremcha, probably of the Black Sea breed, was grey and born in 1941 in Russia, belonging to the Racot Stud. Nakoneczna was born in 1934 at the Łuka Stud and later belonged to the Racot Stud. Another founder mare in Poland was Gurgul V-23, born in 1974 at the Topolčanky Stud in Slovakia. She was brought to the Siary Stud in 1984 as part of an exchange, along with the mare Ousor-6 Adla. Gurgul had full documentation for up to three generations, with one-sided pedigrees tracing back to 1871 (stallion Miszka). Połonina was the founder of the female line through her daughter Polanka and granddaughter Pastuszka, bred by Stanisław Mencel. Sroczka, bred in 1947 in Nowy Sącz County, may have been crossed with a Malopolski horse. Żyrka was a founder through her daughter Sekunda, born in 1956. The mare Agatka was pied, and she may have been named Goral-8, indicating she was the eighth daughter of the Austro-Hungarian stallion Goral. Her first registered daughter was born in 1940. All of these mares, except Gurgul V-23, Sekunda, and Reda, were gathered in 1950 at the Jodłownik Stud, then moved several times, and in 1953 were relocated to Tylicz, and in 1960 to the Siary Stud. The families of these last two mares were maintained for many years only in private breeding [2,8,16].

When comparing results obtained within this study, for families present in the Czech Republic, the Polish Reda family matches completely with the Czech family Dagmar, indicating common ancestry [21]. Reda was born in 1948 in a local breeding in Poland, while other studies [22] describe that the mare Dagmar, born in 1944, was likely a Haflinger-type crossbred, originating from the breeding of Count Johan Palffy in Brezinica, Czech Republic, and was transferred to the SK Topolčany breeding station in Slovakia after World War II (Figure 1). Among dam lines having their representatives in both countries, in the Sroczka family, significant differences were detected in the HVR1B fragment (three nucleotides). A similar situation is observed in the Polanka family, with a six-nucleotide difference in HVR1B. In the Nakoneczna family, two additional polymorphisms were found in the HVR1 fragment. In the maternal line of the Gurgul V-23 mare, there are significant discrepancies (12 nucleotides) when compared to the Slovakian family Aglalia, via mare Gurgul imported to Poland was believed to originate [23]. However, this theory is not supported by a complete pedigree, suggesting that her origin may be different. Alternatively, a similar situation could have occurred with a mare from the Aglalia family imported to the Czech Republic, as the haplotype matches a Hucul horse from Romania [24] without known affiliation to a maternal line. 

Analysis of Hucul horses from Hungary, Austria, and Slovakia compared with Polish Koniks and Przewalski Wild Horse showed that the Czeremcha and Wydra families, haplotypes H and I, may indicate a potential common maternal ancestry [21,25].

Wołga, which originates from Hungary but whose pedigree is unknown, still has a counterpart of its family in Hungary. This family was assigned a haplotype inconsistent with our data, which may indicate errors in the pedigree of the horse brought to Poland (Table 1). The Reda family has its counterpart in Slovakia, which, within the Dagmar family, also occurs in the Czech Republic. The Wydra family also has its counterpart in Hungary, where it originates, as well as in Slovakia; likely, some mare from this family was also registered in the Polish Konik Stud books [21].

It is possible that Czeremcha was not a grey Black Sea pony but rather a light Polish Koniks [8]. However, this hypothesis cannot be considered completely reliable without conducting further studies on a larger segment of mtDNA. The HVR1 fragment shares common maternal ancestry [24]. The Gurgul matriline would share ancestry with Noriker horses, Akhal-Teke horses, and Highland ponies, while Bajkałka and Reda would share ancestry with Sorraia horses. Wołga would have a connection with Romanian Hucul horses and Akhal-Teke horses, and Wydra would also connect with Akhal-Teke horses, Fjord horses, Highland ponies, and Icelandic ponies. If we were to rely solely on HVR1, haplotypes H and I would disappear, which significantly differ in further segments, as would haplotype W2, which is similar to W1 [24]. Therefore, to consider these results reliable and indicative of the founders’ ancestry in different breeds, it would be necessary to expand the research to include additional mtDNA fragments.

Comparing the results with Polish Koniks haplotypes, both Czeremcha and Agatka dam lines completely match the haplotypes of Polish Koniks in the fragment 15,532–15,826. Analyzing only this fragment does not allow for the separation of all the lines of Hucul horses included in this study; however, this fragment itself is common among horses of various breeds and origins [26].

As demonstrated in this study, maternal lines predominantly include mares originating from other lines to a lesser or greater extent. This is the result of numerous errors introduced into the official pedigrees of Hucul horses (Figure 1), likely before mandatory genetic markers inspections for breeding horses. Such errors have also been reported for other horse breeds, such as Arabians, Thoroughbreds, Polish Koniks, and Hucul horses outside Poland [25,26,27,28,29]. It has been suspected that the inconsistencies found in the official pedigrees of Polish Koniks may support the hypothesis that one or more maternal lines, which are commonly believed to no longer exist, are, in fact, still active in the breeding population but are “hidden” due to errors in pedigrees made several generations earlier [26]. A similar situation may occur with Hucul horses, as this analysis also revealed haplotypes that do not match the described families.

The loss of pedigrees after World War II resulted in the unavailability of information regarding the relationships between the founding mares of the lines. The study showed a close relationship between Jagoda and Bajkałka, Sekunda and Sroczka, and a slightly more distant relationship between Reda and Laliszka, as well as Gurgul V-22 and Nakoneczna. However, the Hungarian-origin mares (Wrona, Wołga, and Wydra) were not closely related to each other. Two branches are also visible, separating the families of Wołga, Jagoda, Bajkałka, Reda, and Laliszka from the other horses. This may indicate a distinct ancestry for their ancestors during the initial crossing of Arab, Tatar, Turkish, Przewalski, Nordic, Oriental, and Asian horse breeds.

The study revealed the presence of 17 haplotypes in the mitochondrial DNA of Hucul horses. In addition to the haplotypes that could be assigned to specific maternal lines, three haplotypes were also detected that did not match any family. This may indicate the use of mares that are not recorded in the breed’s studbooks. A discrepancy was also observed between the pedigree affiliation to families and the results of the study. This suggests errors in the pedigrees that occurred before the implementation of mandatory genetic marker testing for breeding Hucul horses. This could have happened, for example, due to accidental substitutions of foals of similar ages among mares kept in groups.

## 5. Conclusions

The results of this study have the potential to improve the genetic management of Hucul horses. The comparison of pedigrees and mtDNA revealed errors in the entries of Hucul horse studbooks. Such information is critical for maintaining genetic diversity and preventing inbreeding. Therefore, studies on maternal line affiliation should be considered mandatory, alongside the already used genetic markers, which only detect errors in recent generations. Additionally, the newly discovered haplotypes found in Hucul horses warrant further examination.

Overall, errors in maternal line pedigrees were identified in 13 horses (22.8%), with no errors found in only 5 out of 14 families (Agatka, Bajkałka, Nakoneczna, Sekunda, and Reda). However, errors could still be detected in a larger sample. Since one of the main objectives of Hucul horse breeding is maintaining a balanced quantitative status of existing families, it is crucial to verify the affiliation of breeding mares to their lines. This may significantly impact the number of mares in individual families and alter the status of families considered endangered. Based on these findings, studbooks should be updated. The increasing matings in primitive horse populations, combined with the limited number of founders and unreliable pedigree information, raise the risk of unintentional inbreeding [30,31].

## Figures and Tables

**Table 1:** (**A**–**C**) Mitochondrial DNA variants located between positions 15,542 and 16,611 detected in study with single nucleotide polymorphisms (SNPs) in all Hucul haplotype sequences compared to the reference sequence NC_001640, with the number of horses belonging to each haplotype included (n).

**A**																														
				**HVR1A**																										
**Haplotype**	**Maternal Line**	**n**	**Accesion Number**	**15,494**	**15,495**	**15,496**	**15,534**	**15,538**	**15,542**	**15,585**	**15,597**	**15,598**	**15,602**	**15,603**	**15,604**	**15,615**	**15,616**	**15,617**	**15,635**	**15,649**	**15,650**	**15,659**	**15666**	**15,667**	**15,703**	**15,709**	**15,720**	**15,726**	**15,740**	
**Reference**		**1**	**NC_001640**	**T**	**T**	**A**	**C**	**A**	**C**	**G**	**A**	**T**	**C**	**T**	**G**	**A**	**A**	**T**	**C**	**A**	**A**	**T**	**G**	**A**	**T**	**C**	**G**	**G**	**A**	
A	Agatka	3	PP002986.1	.	C	.	.	.	.	.	.	C	T	.	.	G	G	.	.	.	.	.	.	.	C	.	A	.	.	
J	Jagoda	3	PP002987.1	.	C	.	.	.	T	A	.	.	T	.	.	.	.	.	.	.	G	.	A	.	.	.	A	.	.	
B	Bajkałka	3	PP002988.1	.	C	.	.	.	T	A	G	.	T	.	.	.	.	.	.	.	G	.	A	.	.	.	A	.	.	
Z	Reda	3	PP002989.1	.	C	.	.	.	T	.	G	.	T	.	.	.	.	.	T	.	G	.	A	.	C	.	A	.	.	
L	Laliszka	8	PP002990.1	.	C	.	.	.	T	.	.	.	T	.	.	.	.	.	T	.	.	.	A	.	C	.	A	.	.	
E	E	3	PP002991.1	.	C	.	.	.	.	.	G	.	T	.	A	.	.	.	T	.	.	.	.	G	C	.	A	.	.	
W1	Wydra	2	PP002992.1	.	C	.	.	.	.	.	.	.	T	.	.	.	.	C	.	.	.	C	.	.	.	.	A	.	.	
W2	Wydra	1	PP002993.1	.	C	.	.	.	.	.	.	.	T	.	.	.	.	C	.	.	.	C	.	.	.	.	A	.	.	
F	Wrona	2	PP002994.1	.	C	.	.	G	.	A	.	.	T	.	.	.	.	.	.	.	.	.	.	.	.	T	A	.	.	
G	Gurgul	3	PP002995.1	C	C	G	T	.	.	A	.	.	.	C	.	.	.	.	.	G	.	.	.	.	.	.	A	.	.	
N	Nakoneczna	4	PP002996.1	C	C	G	T	.	.	A	.	.	T	C	A	.	.	.	.	G	.	.	.	.	.	.	A	.	.	
O	Wołga	7	PP002997.1	.	C	.	.	.	.	.	.	.	T	.	.	.	.	.	.	.	G	.	.	.	.	.	A	.	.	
P	Polanka	4	PP002998.1	.	C	.	.	.	.	.	G	.	T	.	A	.	.	.	T	.	.	.	.	G	C	.	A	.	.	
T	Sekunda	2	PP002999.1	.	C	.	.	.	.	.	.	.	T	.	A	.	.	.	.	.	.	.	.	.	C	.	A	A	G	
S	Sroczka	2	PP003000.1	.	C	.	.	.	.	A	.	.	T	.	A	.	.	.	.	.	.	.	.	.	C	.	A	A	G	
H	H	3	PP003001.1	.	C	.	.	.	.	.	.	.	T	.	.	.	.	C	.	.	.	C	.	.	.	.	A	.	.	
I	I	1	PP003002.1	.	C	.	.	.	.	.	.	.	T	.	.	.	.	C	.	.	.	C	.	.	.	.	A	.	.	
C	Czeremcha	3	PP003003.1	.	C	.	.	.	.	.	.	.	T	.	.	.	.	C	.	.	.	C	.	.	.	.	A	.	.	
**B**																														
		**HVR1B**																												
**Haplotype**	**Maternal Line**	**15,762**	**15,766**	**15,769**	**15,770**	**15,771**	**15,774**	**15,775**	**15,776**	**15,793**	**15,806**	**15,807**	**15,811**	**15,826**	**15,827**	**15,868**	**15,869**	**15,870**	**15,871**	**15,879**	**15,956**	**15,974**	**15,995**	**16,007**	**16,022**	**16,068**	**16,080**	**16,103**	**16,111**	**16,113**
**Reference**		**G**	**C**	**T**	**C**	**C**	**A**	**C**	**T**	**G**	**C**	**C**	**C**	**A**	**A**	**T**	**C**	**C**	**C**	**C**	**A**	**C**	**A**	**T**	**T**	**T**	**G**	**C**	**G**	**G**
A	Agatka	.	.	.	T	.	.	T	.	.	T	.	.	.	G	.	.	.	T	.	G	T	.	.	.	.	.	T	.	.
J	Jagoda	.	.	.	.	T	.	.	.	.	.	.	.	.	.	.	.	T	.	.	G	T	.	.	.	.	.	.	.	A
B	Bajkałka	.	.	.	.	T	.	.	.	.	.	.	.	.	.	.	.	T	.	.	G	T	.	.	.	.	.	.	.	A
Z	Reda	.	.	.	.	.	.	.	.	.	.	.	.	.	.	.	.	T	.	.	.	.	.	.	.	.	.	.	.	A
L	Laliszka	.	.	.	.	.	G	.	.	.	.	.	.	.	.	.	.	T	T	.	.	.	.	.	.	.	.	.	.	A
E	E	A	T	C	T	.	.	T	G	.	.	T	T	.	G	C	.	T	.	.	G	.	G	C	C	.	.	T	A	.
W1	Wydra	A	T	C	T	.	.	T	G	.	.	T	T	.	G	C	.	T	.	.	G	.	G	C	C	.	.	T	A	.
W2	Wydra	A	T	C	T	.	.	T	G	A	.	T	T	.	G	C	.	T	.	.	G	.	G	C	C	.	.	T	A	.
F	Wrona	.	.	.	.	T	.	.	.	.	.	.	.	.	.	.	.	T	.	.	G	T	.	.	.	C	.	T	.	.
G	Gurgul	.	.	.	.	T	.	.	.	.	.	.	.	.	.	.	.	T	T	.	G	T	.	.	.	C	.	T	.	.
N	Nakoneczna	.	.	.	.	T	.	.	.	.	.	.	.	.	.	.	.	T	.	.	G	T	.	.	.	C	.	T	.	.
O	Wołga	.	.	.	.	.	.	.	.	.	.	.	.	G	.	.	.	T	.	.	G	T	.	.	.	.	.	.	.	A
P	Polanka	.	.	.	.	T	.	.	.	.	.	.	.	.	.	.	.	T	T	.	G	T	.	.	.	C	.	T	A	.
T	Sekunda	.	.	.	.	T	.	.	.	.	.	.	.	.	.	.	.	T	.	.	G	T	.	.	.	C	.	T	A	.
S	Sroczka	.	.	.	.	T	.	.	.	.	.	.	.	.	.	.	.	T	.	.	G	T	.	.	.	C	.	T	.	.
H	H	.	.	.	.	.	.	.	.	.	.	.	.	G	.	.	.	.	.	.	.	.	.	.	.	.	.	.	.	A
I	I	.	.	.	.	T	.	.	.	.	.	.	.	.	.	.	.	T	.	.	.	T	.	.	.	C	.	.	.	.
C	Czeremcha	.	.	.	.	T	.	.	.	.	T	.	.	.	G	.	T	.	.	T	G	.	.	.	.	C	A	.	.	.
**C**																														
		**HVR2**																												
**Haplotype**	**Maternal Line**	**16,476**			**16,540**			**16,543**				**16,546**				**16,556**				**IN 16,557:16,558**				**16,559**				**16,629**		
**Reference**		**C**			**C**			**T**				**T**				**T**				**-**				**C**				**A**		
A	Agatka	T			T			A				.				.				-				.				G		
J	Jagoda	.			.			.				.				.				-				.				.		
B	Bajkałka	.			.			.				.				.				-				.				.		
Z	Reda	.			.			.				.				.				-				.				.		
L	Laliszka	.			.			.				.				.				-				.				.		
E	E	.			.			A				.				.				C				.				G		
W1	Wydra	.			.			A				C				.				-				T				G		
W2	Wydra	.			.			A				C				.				-				T				G		
F	Wrona	.			.			.				.				.				C				.				.		
G	Gurgul	.			.			.				.				.				C				.				G		
N	Nakoneczna	.			.			.				.				C				-				.				G		
O	Wołga	.			.			.				.				.				C				.				.		
P	Polanka	.			.			A				.				.				C				.				G		
T	Sekunda	.			.			A				.				.				-				.				G		
S	Sroczka	.			.			A				.				.				-				.				G		
H	H	.			.			A				C				.				-				T				G		
I	I	.			.			A				C				.				-				T				G		
C	Czeremcha	.			.			A				C				.				-				T				G		

•—No differences according to the reference sequence. All obtained haplotypes were deposited in the GenBank database and received accession numbers PP002986.1 to PP003003.1. At Table 1A—HVR1A fragment, at Table 1B—HVR1B fragment, at Table 1C—HVR2 fragment.

## Data Availability

The original sequence of each haplotype presented in the study is openly available in the NCBI database at accession numbers PP002986.1-PP003003.1.

## Supplementary Materials

The following supporting information can be downloaded at: https://www.mdpi.com/article/10.3390/genes15121607/s1, Figure S1. The phylogenetic tree rooted on reference sequence (NC_001640) presents four branches distinguished by color, showing the locations of closely related families. The phylogenetic tree was constructed using MEGA 11 software and edited in PowerPoint.
