# Supplementary material for: Genetic Composition of Polish Hucul Mare Families: mtDNA Diversity
Source: Genes (Basel). 2024 Dec 17;15(12):1607. doi: 10.3390/genes15121607 (PMC11675560; doi:10.3390/genes15121607)
Supplement: Supplementary file 1 [file genes-15-01607-s001.zip › genes-3315255-supplementary.pdf]

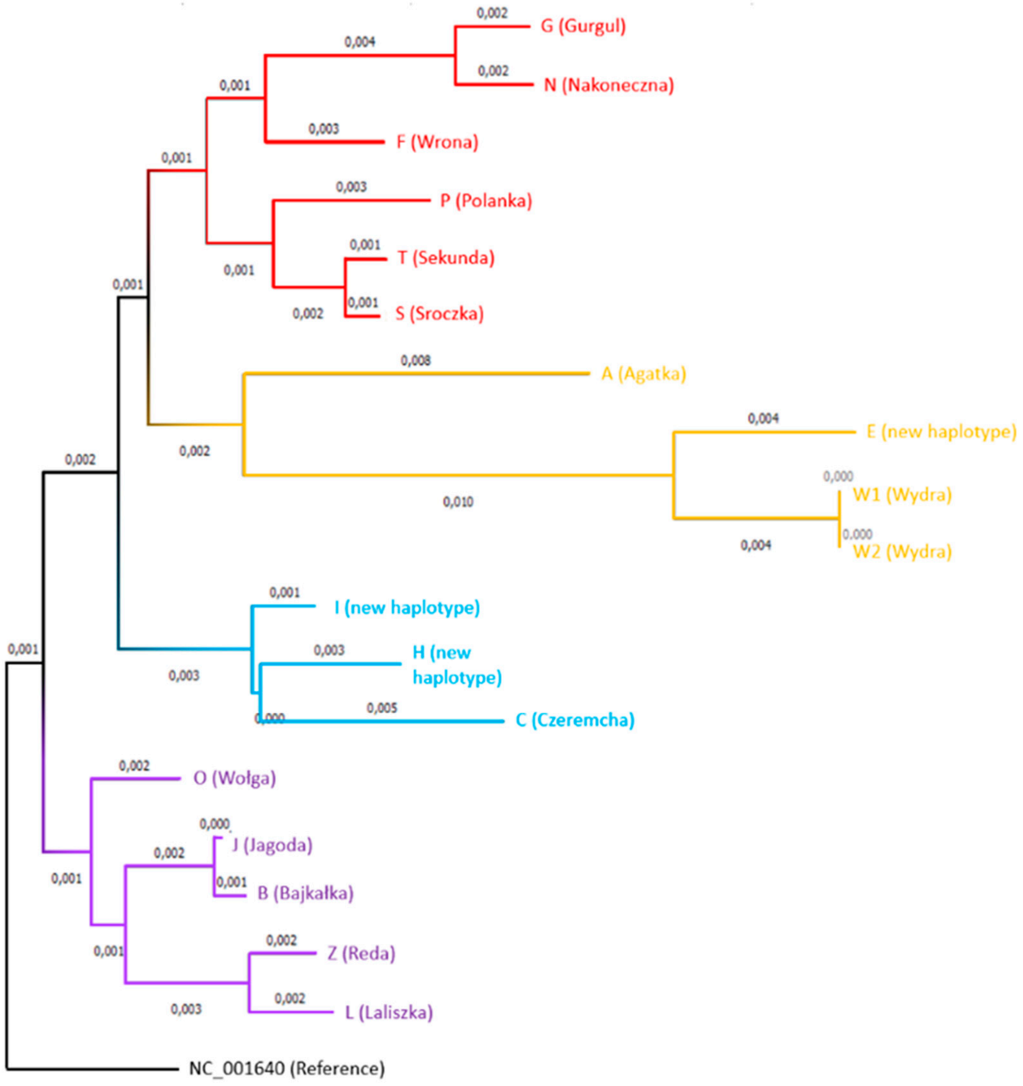

Figure S1. The phylogenetic tree rooted on reference sequence (NC_001640) presents four branches distinguished by color, showing the locations of closely related families. The phylogenetic tree was constructed using MEGA 11 software and edited in PowerPoint.
